# Dexamethasone modulates *Salmonella enterica* serovar Typhimurium infection *in vivo* independently of the glucocorticoid-inducible protein annexin-A1

**DOI:** 10.1111/j.1574-695X.2008.00485.x

**Published:** 2008-09-01

**Authors:** Tomoko Smyth, Sabine Tötemeyer, Sean Haugland, Chrissie Willers, Sarah Peters, Duncan Maskell, Clare Bryant

**Affiliations:** Department of Veterinary Medicine, The University of CambridgeCambridge, UK

**Keywords:** glucocorticoid, dexamethasone, *Salmonella*, infection, annexin-A1

## Abstract

*Salmonella enterica* serovar Typhimurium (*S.* Typhimurium) infection causes an inflammatory response through activation of Toll-like receptor 4 by lipopolysaccharide. Dexamethasone, a glucocorticoid analogue, suppresses inflammatory responses by many mechanisms including inhibition of the lipopolysaccharide-induced production of proinflammatory mediators. There is little information on the effect of glucocorticoids on murine salmonellosis. In this study, we treated susceptible BALB/c mice by subcutaneous implantation of slow-release dexamethasone pellets before infection with *S.* Typhimurium. Dexamethasone promotes bacterial growth early in infection and induces a dose-dependent increase in bacterial growth within mouse livers and spleens. The bacterial load in organs from infected placebo-treated mice was lower than that in dexamethasone-treated mice. Glucocorticoids inhibit lipopolysaccharide-induced inflammation partially through the steroid-inducible protein annexin-A1 (ANXA1). Infection of wild-type and ANXA1 knock-out mice with *S.* Typhimurium led to similar organ bacterial loads. ANXA1 also did not affect the bacterial load in organs from infected dexamethasone-treated mice. This suggests that glucocorticoids, independently of ANXA1, accelerate *S.* Typhimurium growth *in vivo* in susceptible BALB/c mice.

## Introduction

Infection of mice with *Salmonella enterica* serovar Typhimurium (*S.* Typhimurium) causes a systemic disease similar to typhoid fever in humans. The initial bacterial growth rate depends on the genetic background of the mice and the virulence of the bacterial strain. Systemic infection with *S. Typhimurium* results in an initial killing of bacteria, followed by growth of salmonellae in the liver and spleen (Mastroeni & Sheppard, 2004). Host control of early bacterial growth depends on the Nramp/SLC11A1 gene and superoxide production from polymorphoneutrophils. Mice deficient in superoxide production (Mastroeni *et al.*, 2000) or those that have the Nramp-susceptible phenotype, such as BALB/c mice (Fortier *et al.*, 2005), are more susceptible to infection. In sublethal infection, bacterial growth is controlled after several days such that it reaches a plateau phase (Mastroeni, 2002). The induction of this plateau phase depends on the lipopolysaccharide expressed by *S.* Typhimurium activating the pathogen-associated molecular pattern receptor Toll-like receptor 4 (TLR4) in macrophages. This induces an inflammatory response (Weinstein *et al.*, 1986; Khan *et al.*, 1998; Royle *et al.*, 2003; Totemeyer *et al.*, 2005). Lipopolysaccharide-induced activation of TLR4 yields proinflammatory proteins such as cytokines (e.g. tumour necrosis factor α) and inducible enzymes [e.g. inducible nitric oxide synthase (iNOS)] and recruits inflammatory cells, the net result of which is granuloma formation (Muotiala & Makela, 1990; Mastroeni *et al.*, 1991, 1996) with concomitant control of bacterial growth.

Glucocorticoids have many anti-inflammatory effects, including downregulation of proinflammatory mediator production and upregulation of anti-inflammatory proteins. Dexamethasone, a glucocorticoid analogue, blocks the production of many lipopolysaccharide-induced proinflammatory proteins (Waage & Bakke, 1988; Horton *et al.*, 1999; Santini *et al.*, 2001; Korhonen *et al.*, 2002; Li *et al.*, 2002), inhibits superoxide production in guinea-pig alveolar macrophages (Maridonneau-Parini *et al.*, 1989) and reduces cellular migration (Bjornson *et al.*, 1985; Schramm & Thorlacius, 2003; Edwards *et al.*, 2005). Proinflammatory rather than anti-inflammatory (IL4, IL10) cytokine production is preferentially inhibited by dexamethasone shifting the host response to lipopolysaccharide from T-helper type 1 (Th1) to Th2 type (Joyce *et al.*, 1996; Franchimont *et al.*, 1998). This shift in host response is important in mediating the resolution of inflammation, which, if left unchecked, would damage the host. Controversially, the use of steroids at high doses to treat some forms of systemic bacterial infections is being re-evaluated. Administration of glucocorticoids, for example, while maintaining the circulation, can exacerbate infection (Prigent *et al.*, 2004). Steroid treatment regimes need to be designed carefully to limit tissue-damaging inflammatory responses, but not to enhance infection; therefore, a clear understanding of how glucocorticoids affect the growth of bacteria *in vivo* is required. There is limited published work on the effects of glucocorticoids on *S.* Typhimurium infection *in vivo*. Earlier work by Hobson (1960) showed that pretreatment of mice with single high doses of cortisone increased bacteraemia, increased bacterial proliferation, prevented the plateau phase and decreased the time to death after intravenous administration of *S.* Typhimurium. In chronic murine salmonellosis, mortality was increased by glucocorticoid treatment (Plant *et al.*, 1983).

Annexin-A1 (ANXA1) is a glucocorticoid-regulated protein that is required for some, but not all, the anti-inflammatory actions of glucocorticoids (Perretti & Flower, 2004). These include the reduction of oedema, a decrease in polymorphoneutrophil cell migration, inhibition of pyrexia and inhibition of some of the effects of lipopolysaccharide (Wu *et al.*, 1995; Perretti & Flower, 2004). N-terminal peptides of ANXA1 modify the inflammatory responses of leucocytes (Perretti, 1998) and ANXA1 modulates lipopolysaccharide induction of iNOS expression (Wu *et al.*, 1995; Bryant *et al.*, 1998; Smyth *et al.*, 2006). ANXA1 peptidomimetics are under development to treat conditions such as septic and endotoxic shock, and ANXA1^−/−^ mice are more susceptible to endotoxaemia (Damazo *et al.*, 2005). The effect of ANXA1 on the host response to infection is unknown.

Glucocorticoids, therefore, inhibit many lipopolysaccharide-induced responses, but studies on the effects of glucocorticoids on the progression of *Salmonella* infections *in vivo* are sparse (Hobson, 1960; Katler & Weissmann, 1977). The role of ANXA1 in modifying the host response to lipopolysaccharides suggests that this protein might either decrease the inflammatory response of the host to *S.* Typhimurium infection or modulate some of the effects of glucocorticoids during infection. Here, we investigated whether the synthetic glucocorticoid dexamethasone, when administered chronically to susceptible BALB/c mice, modified the host response to *S.* Typhimurium and whether ANXA1 is important in this process.

## Materials and methods

### Reagents, cells and animals

Dexamethasone and placebo pellets for implantation were purchased from Innovative Research of America, Sarasota, FL. Six- to eight-week-old BALB/c mice were purchased from Harlan, UK. ANXA1^−/−^ and ANXA1^+/+^ mice on a BALB/c background (Hannon *et al.*, 2003) were a gift from Prof. R. Flower (Queen Mary College, University of London).

### Dexamethasone administration *in vivo*

All dexamethasone and placebo pellets used were designed for a 21-day continuous release of dexamethasone (Innovative Research of America). The total dexamethasone content of the pellets used was 0.5 mg or 1.5 mg per pellet. These are equivalent to dose levels of 1.2 and 3.6 mg kg^−1^ day^−1^, respectively, in a 20 g animal. Each animal was subcutaneously implanted with one pellet under general anaesthesia induced by isoflurane (Abbott Laboratories, Queenborough, UK). Animals were allowed to recover for 2 days before further procedures were undertaken.

### Plasma dexamethasone measurement

The dexamethasone content in plasma was confirmed using a dexamethasone enzyme immunoassay (EIA) kit according to the manufacturer's protocol (Bio-X, Germany), using dexamethasone diluted in normal mouse plasma to generate a standard curve.

### *In vivo S.* Typhimurium infection

*Salmonella* M525P causes a sublethal infection in Nramp-susceptible mouse stains such as BALB/c and C57Bl/6 (Hormaeche, 1979). M525P was grown statically overnight in 10 mL of Luria–Bertani (LB) broth. Bacteria were harvested by centrifugation and resuspended in 10 mL of sterile phosphate-buffered saline (PBS). The absorbance was measured to estimate the CFU mL^−1^. The bacterial suspension was diluted to 5 × 10^3^ CFU mL^−1^ in PBS and 0.2 mL was used to inoculate each animal intravenously (10^3^ CFU per animal). The bacterial inoculum was plated out on LB agar and the bacterial number was estimated. Animals were killed at various time points (4 h–14 days subject to the condition of mice) after infection, the livers and spleens were removed and divided for different uses. One organ section was homogenized in 10 mL of sterile water, and appropriate dilutions were plated onto LB agar plates for viable bacterial counts. Organ sections were also either snap-frozen in liquid nitrogen to isolate total RNA or fixed in 10% formalin for histological analyses. Blood was collected by a cardiac puncture into heparinized syringes to prepare plasma samples. Blood smears were prepared for differential white blood cell counts, and 200 white blood cells were counted. In a separate study, mice were infected intravenously with 1 × 10^6^ CFU mL^−1^ of *S.* Typhimurium and serial blood samples were taken from the retrobulbar vein. Blood samples were diluted in sterile water and plated onto LB agar plates to count viable bacteria. All mouse studies were conducted in accordance with Home Office welfare and ethical regulations.

### mRNA analyses

iNOS mRNA was detected by reverse transcriptase (RT)-PCR using the following oligonucleotides: forward primer, 5′-CGCAGCTGGGCTGTACAA-3′; reverse primer, 5′-TGATGTTTGCTTCGGACATCA-3′, and probed with 5′-CTTCCGGGCAGCCTGTGAGACCT-3′. ANXA1 mRNA was detected using the following oligonucleotides: forward, 5′-TTTGCCGAGAAGCTGTACGA-3′; reverse, 5′-CGAACGGGAGACCATAATCCT-3′; and probe, 5′-CCGGAACTCGCCATAAGGCATTGA-3′. mRNA of TLR4, TLR2, MD-2 and 18S RNA gene were quantitated by real-time PCR as described (Hormaeche, 1979; Totemeyer *et al.*, 2003, 2005). All probes were dual labelled with 5′FAM and 3′BHQ-1 (Corbett Research, UK). Real-time RT-PCR was performed and analysed on a RotorGene3000 machine (Corbett Research) with oligonucleotides purchased from Operon (UK) and probes purchased from Eurogentec (UK). Fold differences in mRNA values were calculated as two to the power of (40−*C*_t_ of test)−(40−*C*_t_ of control).

### Histology

Liver and spleen samples were fixed in 10% formalin for at least 24 h. The sections were embedded in paraffin and 5-μm sections were cut and stained with haematoxylin–eosin. A Nikon E400 microscope with an eyepiece graticule was used to count polymorphoneutrophils and macrophages of one or two serial sections from each tissue (equivalent to an area of 1.15 mm^2^), the cells being counted under a × 20 high power (× 40 objective). Two separate measurements were made: the total number of macrophages and polymorphoneutrophils per field, and the number of macrophages and polymorphoneutrophils within recognizable lesions only.

### Statistical analysis

Statistical analyses were carried out using prism 3.0 (Graphpad). Student's (unpaired) *t*-test was used for comparison of two groups. One-way anova (with Dunnet's post-test where appropriate) was used for comparison of more than two groups. Differences were considered to be statistically significant when *P*<0.05.

## Results

### Effects of dexamethasone on the haematological profile and spleen weight of uninfected animals

**Fig. 1:**
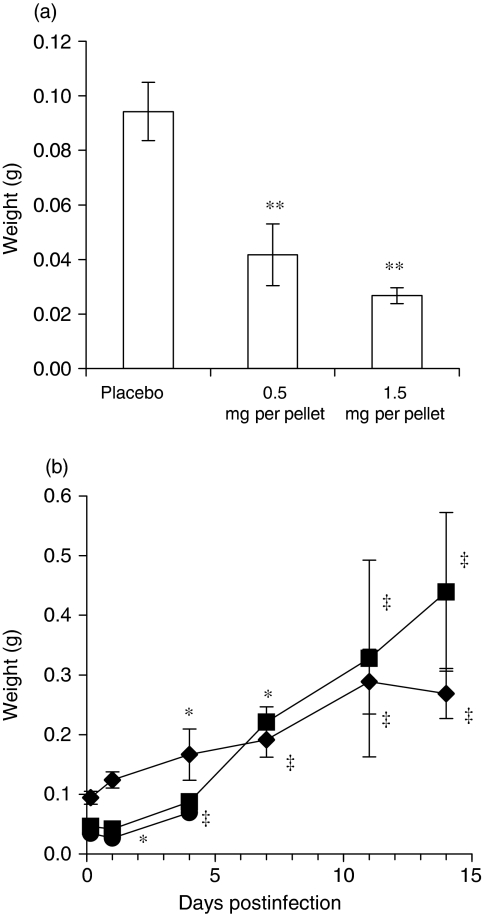
Effect of dexamethasone on the spleen weight of mice. (a) Spleen weights of mice implanted with placebo, 0.5 or 1.5 mg kg^−1^ dexamethasone pellets 2 days postimplantation (the statistical significance compared with the placebo-treated group is indicated by ^**^P<0.01). (b) Spleen changes in mice implanted (subcutaneously) with placebo (♦) or 0.5 mg per pellet (▪) or 1.5 mg per pellet (•) of dexamethasone and infected intravenously with 10^3^ bacteria. Spleens were weighed at regular time points after infection. Data are expressed as mean weight (g)±SD of at least three mice per time point per treatment, and are representative of two independent experiments. Statistically significant increases in spleen weights compared with day 0 are indicated by ^*^P<0.05; ^**^P<0.01 and ^‡^*P*<0.001. Spleen weights were significantly increased to a similar degree in both the placebo and the dexamethasone groups of infected mice compared with the uninfected mice from day 4 of the infection onwards.

Plasma levels of dexamethasone were measured by EIA after implantation of the slow-release pellets. The EIA indicated a low level of dexamethasone in the serum of placebo-treated animals (1.59±0.98 ng mL^−1^), presumably due to cross-reactivity to endogenous cortisone. Dose-dependent levels of dexamethasone were present, as expected, in animals that received the dexamethasone implants (0.5 or 1.5 mg per pellet). Dexamethasone levels were elevated above the baseline level for up to 13 days postimplantation. In infection experiments, the levels of dexamethasone (ng mL^−1^ mean±SD) in infected mice implanted with 0.5 mg kg^−1^ pellet were 23.69±20.19 at day 4 (day 6 postimplantation), 13.22±5.13 at day 7 (day 9 postimplantation) and 18.78±13.56 ng mL^−1^ at day 13 (day 15 postimplantation). In infected mice implanted with the 1.5 mg kg^−1^ pellet, plasma levels of dexamethasone were 76.22±59.63 ng mL^−1^ at day 4 of the infection (day 6 postimplantation). To verify dexamethasone efficacy, we measured the relative polymorphoneutrophil count in blood and spleen size throughout infection. In blood smears, the relative numbers of blood polymorphoneutrophils were elevated in dexamethasone-treated animals (36.0±9.8% for 0.5 mg per pellet and 87.3±0.6% for 1.5 mg per pellet) compared with placebo-treated animals (19.0±5.6%) 3 days after pellet implantation. The relative number of circulating lymphocytes decreased from 78.3±7.6% in placebo-treated mice to 12.3±0.6% in 1.5 mg per pellet dexamethasone-treated animals. Dexamethasone, therefore, induced a classical haematological profile at both doses used in this study. Dexamethasone causes a reduction in spleen weight *in vivo* (Rooman *et al.*, 1999). Animals that received 0.5 or 1.5 mg dexamethasone per pellet had spleens that were less than half the weight of the placebo-treated animals 2 days after pellet implantation (Fig. 1a).

### Effects of dexamethasone on the control of *S.* Typhimurium infection

**Fig. 2:**
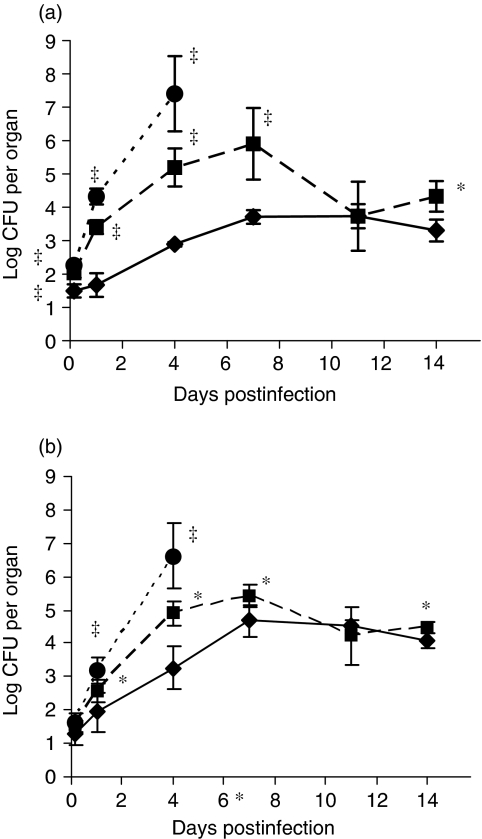
Effects of dexamethasone on the growth of Typhimurium in the liver (a) and spleen (b) of Balb/c mice. Mice were implanted (subcutaneously) with placebo (♦) or 0.5 mg per pellet (▪) or 1.5 mg per pellet (•) of dexamethasone and infected intravenously with 10^3^ bacteria. Liver or spleen bacterial counts were taken at regular time points post infection. Data are expressed as the mean CFU per organ±SD of at least three mice per time point, and are representative of two independent experiments. Statistical significance between dexamethasone- and placebo-treated mice is indicated on the graph (^*^P<0.05; ^‡^*P*<0.001).

In placebo-treated animals, as expected, there was little growth of *S.* Typhimurium during the first 4 h of infection in livers (Fig. 2a) or spleens (Fig. 2b). This initial lag phase in bacterial growth was reduced in livers from animals treated with either 0.5 or 1.5 mg per pellet dexamethasone (Fig. 2a; *P*<0.001 vs. placebo treated). The bacterial load was higher in the liver at both doses of dexamethasone in comparison with the placebo-treated infected mice (Fig. 2a). Bacterial load was also increased in the spleen, but this only reached statistical significance between placebo-treated and 1.5 mg per pellet dexamethasone-treated mice on day 4 (Fig. 2b). The dexamethasone-induced bacterial growth rate slowed between days 1 and 4 in the 0.5 mg per pellet-treated mice, but not in the 1.5 mg per pellet-treated mice (Fig. 2b). The organ bacterial loads for both dexamethasone-treated groups of animals were significantly higher than the bacterial load of log 3–log 4 CFU per organ observed in placebo-treated animals on day 4. At the higher dose of dexamethasone, mice could not control bacterial growth and therefore the experiment was stopped in this group of animals. The bacterial numbers in the mice treated with the 0.5 mg per pellet of dexamethasone started to decrease after day 7, showing that at this dose of dexamethasone, these mice were able to control the infection.

To determine whether dexamethasone was affecting bacterial clearance from the blood on day 1, we measured the bacterial levels in blood up to 4 h after infecting with *S.* Typhimurium. We observed no difference in the bacterial clearance rate between the placebo-treated mice and those treated with both doses of dexamethasone. All bacteria had been cleared from the bloodstream within 4 h of infection (data not shown) in all mice. A low level of bacteraemia was observed in mice treated with the 1.5 mg of dexamethasone pellet on day 4 postinfection (mean±SD of log 5.3±log 5.2 CFU mL^−1^ blood; the large SD is because some animals had no bacteraemia). No bacteraemia was observed in infected animals treated with the 0.5 mg dexamethasone pellet.

### Effects of dexamethasone on spleen and liver infected with *S.* Typhimurium

Typhimurium induces an inflammatory response in the host that is associated with splenomegaly. There were no significant differences between the spleen weights of infected and uninfected animals on days 0 and 1 postinfection. Typhimurium infection induced splenomegaly in placebo-treated mice. On days 11 and 14, spleen sizes were 3.1 and 2.9 times the size of the spleens on day 0 of infection (Fig. 1b). Although the low-dose dexamethasone pellet (0.5 mg) reduced spleen size by 43±10% before infection, it did not inhibit *S.* Typhimurium from inducing splenomegaly (Fig. 1). In fact, splenomegaly was more pronounced in these mice, with the mean spleen weights on days 11 and 14 reaching 3.5 and 4.7 times the spleen sizes of placebo-treated mice on day 0. This is equivalent to 7.1 and 9.5 times the mean size of spleens in the dexamethasone-treated mice before infection. Typhimurium infection also partially reversed the decrease in spleen size induced by 1.5 mg per pellet dexamethasone treatment on day 4.

**Fig. 3:**
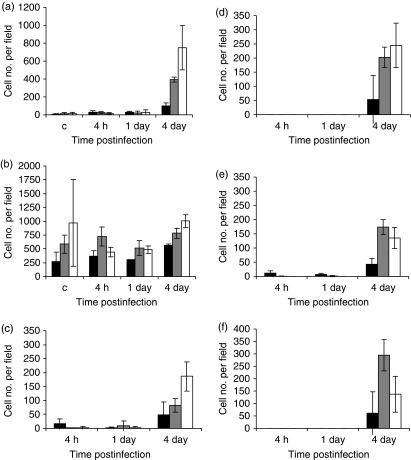
Effects of dexamethasone on the macrophage and polymorphoneutrophil population in the liver and spleen of mice implanted (subcutaneously) with placebo (black bar) or 0.5 mg per pellet (grey bar) or 1.5 mg per pellet (white bar) of dexamethasone and infected with 10^3^ Typhimurium. (a) Total polymorphoneutrophils in the liver, (b) total polymorphoneutrophils in the spleen, (c) granuloma-associated, polymorphoneutrophils in liver, (d) granuloma-associated polymorphoneutrophils in spleen, (e) granuloma-associated macrophages in the liver and (f) granuloma-associated macrophages in the spleen. Cells were counted in a 1.15 mm^2^ field. c, control.

The numbers of polymorphoneutrophils during Typhimurium infection were increased in lesions in both the spleen and the liver on day 4 in all treatment groups (Fig. 3a and b). More cells were recruited to infected organs in dexamethasone-treated groups compared with the placebo group. There was no difference in the total spleen polymorphoneutrophils in placebo-treated or either of the dexamethasone-treated groups, probably because of the large individual variation within the group (Fig. 3b). Polymorphoneutrophil numbers in livers up to day 1 were too low for accurate comparison. However, on day 4, dexamethasone treatment resulted in an increase in the total polymorphoneutrophils in the liver in comparison with the infected placebo-treated animals. In both organs, the number of polymorphoneutrophils associated with granulomata increased by day 4 (Fig. 3c and d). The number of macrophages associated with granulomata in the spleen and liver also increased in 0.5 mg per pellet dexamethasone-treated animals (Fig. 3e and f). The apparent lack of increase in macrophage association with granulomata in the 1.5 mg per pellet dexamethasone-treated animals may be due to the inhibitory effects of high doses of dexamethasone on cell migration.

### Effects of dexamethasone on gene expression induced by infection of mice with *S.* Typhimurium

**Fig. 4:**
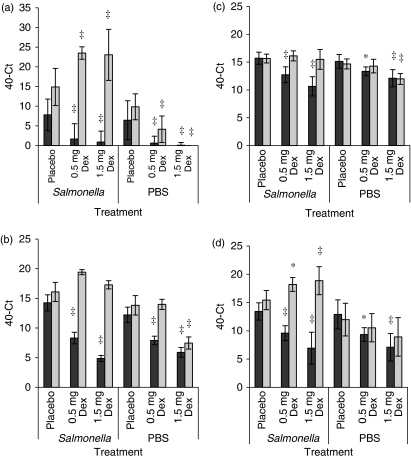
Real-time RT-PCR analyses of iNOS (a), TLR4 (b), MD2 (c) and TLR2 (d). mRNA levels in the liver of Typhimurium-infected mice with or without dexamethasone. Animals were killed on day 1 (black bar) and day 4 (grey bar) postinfection. Data are expressed as mean±SD of at least six mice per time point [*C*_t_ (cycle time) subtracted from 40 (the negative end-point)]. Higher values represent higher levels of mRNA. Statistical significance between dexamethasone- and placebo-treated mice is indicated on the graph (^*^P<0.05; ^‡^*P*<0.001).

Dexamethasone suppresses inflammatory gene expression. We therefore investigated whether dexamethasone altered *S.* Typhimurium-induced proinflammatory gene expression using quantitative RT-PCR from total liver RNA. In uninfected placebo-treated animals, iNOS mRNA was basally expressed, but basal iNOS expression was suppressed in dexamethasone-treated mice. Typhimurium infection increased iNOS expression sixfold in placebo-treated mice between day 1 and day 4 of infection (*P*<0.01). A differential response in iNOS expression was revealed between day 1 and day 4 after infection in dexamethasone-treated mice. Dexamethasone inhibited both basal and *S.* Typhimurium-stimulated iNOS expression at day 1, but by day 4 there was a significant potentiation in iNOS mRNA expression in response to infection (Fig. 4a). The expression of iNOS mRNA in infected dexamethasone-treated mice was increased by *c*. 3.7 × 10^6^-fold in the 0.5 mg per pellet group and by 4.6 × 10^6^-fold in the 1.5 mg per pellet groups from day 1 to day 4.

Glucocorticoids modulate TLR expression; hence, we investigated whether the elevated iNOS expression observed on day 4 of infection in dexamethasone-treated mice was due to enhanced TLR4 expression causing an increased sensitivity of the mice to *S.* Typhimurium lipopolysaccharide. Dexamethasone treatment reduced the expression of TLR4, MD-2 and TLR2 mRNA in uninfected animals in a dose-dependent manner (Fig. 4b–d). In placebo-treated animals, the levels of TLR4 mRNA remained relatively unchanged during infection, but TLR4 mRNA expression was still decreased in animals of both dexamethasone-treated groups by day 1 postinfection. By day 4 postinfection, the levels of TLR4 mRNA in the dexamethasone-treated animals were the same as those in the placebo-treated infected animals, indicating that there was a greater increase in mRNA in dexamethasone-treated animals between days 1 and 4 than in placebo-treated animals. In the dexamethasone-treated infected animals, MD2 mRNA levels decreased on day 1 postinfection, but were subsequently restored to levels similar to those of the infected placebo-treated animals. TLR2 mRNA levels were decreased transiently by both doses of dexamethasone in uninfected animals. Infection enhanced TLR2 mRNA in all groups of mice by day 4. The upregulation in TLR4, MD-2 and TLR2 mRNA by day 4 of *S.* Typhimurium infection coincides with the upregulation of iNOS mRNA and with the influx of inflammatory cells into the liver. Inflammatory cell influx into the liver is likely to account for some, but not all, the changes in gene expression that we observed (Totemeyer *et al.*, 2003, 2005).

### Effects of ANXA1 on the host response to *S.* Typhimurium infection in the presence or absence of dexamethasone

**Fig. 5:**
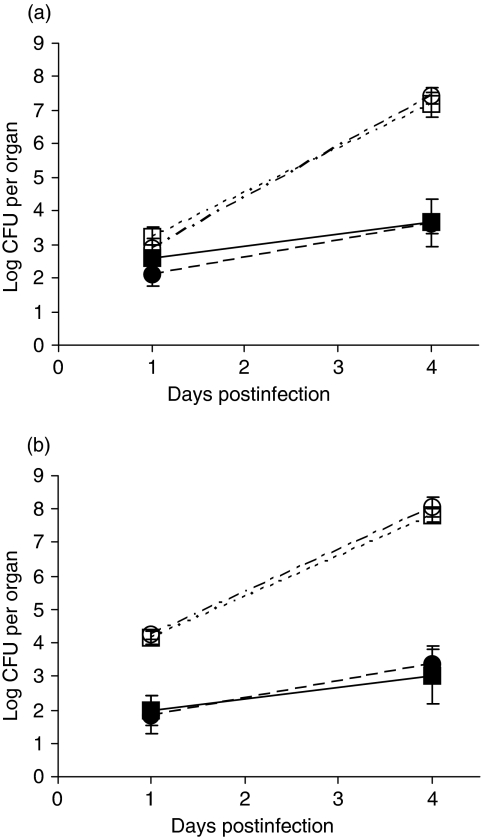
Growth of Typhimurium in the spleen (a) and liver (b) of ANXA^+/+^ (square) and ANXA1^−/−^ (circle) mice with (open) or without (filled) dexamethasone treatment. The data are mean±SD of at least three animals per time point.

ANXA1^−/−^ and ANXA1^+/+^ mice were injected intravenously with log 3 CFU of *S.* Typhimurium strain M525P or PBS. The *S.* Typhimurium growth rates were identical in both ANXA1^+/+^ and ANXA1^−/−^ mice strains (Fig. 5), indicating that ANXA1 does not influence the host immune response to *S.* Typhimurium infection. When ANXA1^+/+^ and ANXA1^−/−^ animals were implanted with either placebo or 1.5 mg dexamethasone pellets, and subsequently infected with *S.* Typhimurium, bacterial growth was enhanced by dexamethasone to a similar extent in both strains of mice (Fig. 5). Spleens from both strains of uninfected mice were similar in weight. The effects of dexamethasone on spleen weight in both uninfected and infected mice were independent of ANXA1 (data not shown). Dexamethasone, therefore, had similar effects on *S.* Typhimurium infection in both ANXA1^+/+^ and ANXA1^−/−^ mice, suggesting that ANXA1 is unlikely to be important in mediating the effects of glucocorticoids during the early phase of this infection.

## Discussion

In this study, we found that the synthetic glucocorticoid analogue, dexamethasone, dose dependently suppressed the ability of susceptible mice to control *S.* Typhimurium infection. Glucocorticoids suppress inflammation, and an inflammatory response to *S.* Typhimurium is critical for the successful control of bacterial growth (Khan *et al.*, 2001). The glucocorticoid-inducible protein ANXA1 mediates some, but not all, the anti-inflammatory effects of glucocorticoids (Flower, 1990). Here, ANXA1 had no effect on *S.* Typhimurium infection in the presence or absence of glucocorticoids. Studies in ANXA1^−/−^ mice show an important role for ANXA1 in protecting animals against the effects of inflammation and systemic lipopolysaccharide administration (Hannon *et al.*, 2003; Damazo *et al.*, 2005), and ANXA1^−/−^ macrophages show deficits in phagocytic activity *in vitro* (Yona *et al.*, 2004). Lipopolysaccharide activation of TLR4 plays a key role in driving the protective immune response against *S.* Typhimurium infection (Weinstein *et al.*, 1986; Royle *et al.*, 2003; Totemeyer *et al.*, 2003; Weiss *et al.*, 2004). We, therefore, expected ANXA1^−/−^ mice infected with *S.* Typhimurium to show an altered response to infection with *S.* Typhimurium *in vivo*. ANXA1 also did not play a role in mediating the effects of dexamethasone on *S.* Typhimurium infection in our study. This suggests a differential effect of ANXA1 on inflammation induced by lipopolysaccharide and infection with *S.* Typhimurium. While this is unexpected, given the importance of lipopolysaccharide in driving a protective immune response against *S.* Typhimurium infection, perhaps it is not so surprising, given the range of other bacterial factors, such as bacterial proteins, that modify host inflammatory signalling, produced by *Salmonella* species. Our data suggest that ANXA1 plays no role in the pathogenesis of infection and it may be that peptides based on ANAX1 could therefore be useful immunomodulators in infected patients.

Dexamethasone, as expected, had a marked effect on *S.* Typhimurium infection *in vivo*. The increased growth of *S.* Typhimurium seen in dexamethasone-treated animals is consistent with earlier studies conducted by Hobson in 1960 using single bolus injections of cortisone in different strains of mice infected with *S.* Typhimurium (Hobson, 1960). Here, we used dexamethasone as a slow-release implant to minimize the handling of animals and the consequent stress-induced release of endogenous glucocorticoids. Administration of dexamethasone via subcutaneous implants produced a classical steroid profile in the mice, including neutrophilia and a reduction in spleen weight. Our data show that dexamethasone dose dependently increases bacterial load by day 1 of infection. Dexamethasone suppresses the production of many inflammatory proteins including TNFα (Ogawa *et al.*, 2005) and iNOS (Korhonen *et al.*, 2002). Suppression of TNFα, IFNγ, iNOS, IL-12 or IL-18 increases the host susceptibility to *S.* Typhimurium (Nauciel & Espinasse-Maes, 1992; Mastroeni, 2002), but these proteins are not involved in controlling the initial bacterial growth rate. The initial killing of *S.* Typhimurium is mainly driven by nicotinamide adenine dinucleotide phosphate oxidase (phox) and Nramp activity (Mastroeni, 2002). Dexamethasone does not affect the expression of cytochrome b-245 (p22-phox) (Ogawa *et al.*, 2005), but does suppress phagocytic oxidative burst activity (Roshol *et al.*, 1995). Cortisone enhances the growth of mycobacteria in macrophages and suppresses Nramp expression in susceptible mice, such as the BALB/c strain used in this study (Brown *et al.*, 1995). To determine the precise mechanism of how corticosteroids affect *S.* Typhimurium growth *in vivo*, more experiments need to be performed, particularly comparing mouse strains with functional and nonfunctional Nramp, to determine the importance of phox activity in the dexamethasone-induced increase in the bacterial growth rate.

Bacterial growth was controlled after day 7 postinfection in 0.5 mg dexamethasone-treated animals, and bacteria started to be cleared. The check in bacterial growth is unlikely to be due to a decline in the serum dexamethasone level because this did not occur until after day 11 of the infection and the biological half-life of dexamethasone can be as long as 48 h. At the higher dose of dexamethasone, no check in bacterial growth was apparent, but this is because the high bacterial load seen in these animals led to an early termination of the experiment. Our observations suggest that dexamethasone either may not inhibit or only has an effect at high doses on the bacterial clearance mechanisms involved in the later stages of infection.

Dexamethasone alters the distribution of leucocytes and prevents the induction of many chemokines and, therefore, we were interested in how dexamethasone might influence the host response to infection. Dexamethasone increased the total numbers of polymorphoneutrophils and macrophages profoundly in animals infected with *S.* Typhimurium by day 4 of infection. The polymorphoneutrophil count increased in a dose-related manner, and this is probably associated with the direct effect of dexamethasone on polymorphoneutrophil migration and with the increased bacterial burden seen in infected animals with increasing doses of the drug. The liver of infected animals treated with the highest dose of dexamethasone showed that the bacteria were present not only in polymorphoneutrophils and macrophages but also in hepatocytes, again probably due to the increased bacterial load in these animals. Dexamethasone suppresses lipopolysaccharide-induced polymorphoneutrophil and macrophage migration (Perretti, 1998), but does not appear to suppress polymorphoneutrophil migration into lesions in response to *S.* Typhimurium infection *in vivo*.

The suppression of inflammatory gene expression by glucocorticoids is well known and, indeed, we showed iNOS mRNA suppression by dexamethasone in uninfected animals. Yet, in our study, by day 4 of infection *S.* Typhimurium-induced iNOS mRNA expression was greater in dexamethasone-treated animals than in the placebo-treated mice. This was accompanied by enhanced upregulation in mRNA for TLR4, MD-2 and TLR2 (proteins that mediate lipopolysaccharide-induced iNOS transcription) in dexamethasone-treated mice in comparison with placebo-treated animals. Glucocorticoids modulate TLR expression, although precisely how this occurs is unclear. Sakai *et al.* (2004) reported that TLR2 expression in epithelial cells is enhanced by dexamethasone, and speculated that dexamethasone enhances the host defence against bacterial infection by upregulating TLR2. Ogawa *et al.* (2005) found no evidence for the glucocorticoid regulation of TLR expression, whereas Rozkova *et al.*, (2006) showed enhanced TLR2 and TLR4 expression accompanied by decreased dendritic cells maturation in patients treated with high levels of glucocorticoids. Dexamethasone reduces inflammatory cytokine production from peripheral blood mononuclear cells infected with *Streptococcus pneumoniae* or *Neisseria meningitidis* by inhibiting the activation of the transcription factor nuclear factor κB, but glucocorticoid does not affect the increased mRNA stability induced by bacterial infection (Mogensen *et al.*, 2008). Here, we measured TLR expression in the whole organ over a long course of time. It is quite likely that our results are complicated by the presence of many cell types within the liver with a differential sensitivity to regulation by glucocorticoids and also by the opposing effects of dexamethasone and bacteria on gene mRNA transcription and stability, respectively. The differences we see in TLR levels and iNOS transcription in response to dexamethsone *in vivo* therefore need further investigation.

In conclusion, the glucocorticoid analogue dexamethasone suppresses the ability of the susceptible mice to control *S.* Typhimurium infection, particularly at the early innate resistance phase, suggesting that this compound is inducing defects in the cellular bacterial killing mechanisms. The effects of glucocorticoids are independent of ANXA1, suggesting that while this protein modulates some of the effects of exogenously administered lipopolysaccharide, it does not affect the host response to infection with *S*. Typhimurium.

## Statement

Re-use of this article is permitted in accordance with the Creative Commons Deed, Attribution 2.5, which does not permit commercial exploitation.
